# Factors associated with prolonged non-nutritive sucking habits in two cohorts of Brazilian children

**DOI:** 10.1186/1471-2458-14-743

**Published:** 2014-07-22

**Authors:** Marcela Maia-Nader, Camilla Silva de Araujo Figueiredo, Felipe Pinheiro de Figueiredo, Antônio Augusto Moura da Silva, Erika Bárbara Abreu Fonseca Thomaz, Maria Conceição Pereira Saraiva, Marco Antonio Barbieri, Heloisa Bettiol

**Affiliations:** 1 Department of Puericulture and Pediatrics, Ribeirão Preto Medical School, University of São Paulo, Avenida Bandeirantes 3900, Zip Code 14049-900 Ribeirão Preto, São Paulo, Brazil; 2 Department of Public Health, Federal University of Maranhão, São Luís, Maranhão, Brazil; 3 Department of Neurosciences, Ribeirão Preto Medical School, University of São Paulo, Ribeirão Preto, São Paulo, Brazil; 4 Department of Pediatrics and Social Dentistry, Faculty of Dentistry of Ribeirão Preto, University of São Paulo, Ribeirão Preto, São Paulo, Brazil

**Keywords:** Non-nutritive sucking habits, Finger sucking, Pacifier sucking, School age children

## Abstract

**Background:**

Non-nutritive sucking habits (NNSH) are very common during childhood. However, if these habits were maintained for 36 months of age or more, they are considered to be prolonged (PNNSH) and can cause occlusal, physiological and esthetic changes. There is controversy about their prevalence and whether perinatal, social, demographic and health characteristics influence their onset and duration. So, the objectives of this study are to estimate the prevalence of PNNSH and to evaluate perinatal, early life and school age factors associated with their occurrence in children.

**Methods:**

A sample of 1,463 children aged 7–11 years born in Ribeirão Preto (RP-1994) and São Luís (SL-1997/98), Brazil, was reevaluated at school age in 2004/05. Birth weight, gestational age and perinatal variables were obtained at birth. Type of feeding, occurrence and duration of finger and pacifier sucking were recorded retrospectively at school age. PNNSH were defined when persisted for 36 months of age or more. Crude and adjusted prevalence ratios (PR) were estimated by Poisson regression (alpha = 5%).

**Results:**

Prevalence of PNNSH was higher in RP (47.6%) than in SL (20.2%) – (p < 0.001). Perinatal variables were not associated to PNNSH, whilst female sex (PR = 1.27 in RP; PR = 1.47 in SL) and bottle feeding for 24 months or more (PR = 2.24 in RP; PR = 2.49 in SL) were risk factors in both locations. Breast feeding for 12 months or more (PR = 0.53 in RP; PR = 0.31 in SL) was associated with lower prevalence of PNNSH in both places. In SL, children whose mothers lived in consensual union (PR = 1.62) and worked outside the home (PR = 1.51) showed higher prevalence of PNNSH compared to their counterparts.

**Conclusions:**

Prevalence of PNNSH was high especially in RP and was not associated with perinatal variables. In both cities there was an association between female sex, shorter breast-feeding duration, longer bottle feeding duration and higher prevalence of PNNSH.

## Background

Sucking habits are actions acquired by the frequent repetition of conscious or unconscious neuromuscular activities regulated by reflex arches originating from psychological needs [1,2]. They are considered to be nutritive when they satisfy nutritional needs and non-nutritive when they satisfy psychological needs [3,4].

Non-nutritive sucking habits (NNSH) are very common during childhood [5,6]. However, NNSH can cause occlusal, physiological and esthetic changes [7,8], especially if maintained for 36 months of age or more, when they are considered to be prolonged–PNNSH [9].

Breast-feeding has been associated with a lower occurrence of these habits [10-13], whereas the use of a bottle seems to be related to their development and persistence [11-14]. Biological and social factors are also associated with PNNSH [5,15,16]. However, there is controversy about whether preterm birth, birth weight and maternal age at childbirth influence the onset and duration of these habits.

Most of the studies on this subject have been conducted on small, convenience samples with a cross-sectional design and with little or no control for confounding factors [9,11,12,16]. The objective of the present study was to estimate the prevalence of PNNSH (finger and pacifier) and to evaluate perinatal, early life and school age factors associated with their occurrence in children belonging to two Brazilian birth cohorts.

## Methods

The data used were from two cohort studies [17,18] conducted on liveborn individuals in the municipalities of São Luís (SL) in 1997/98, and Ribeirão Preto (RP), in 1994. RP is a wealth and industrialized city in the interior of São Paulo State located in the Southeast region of the country, with a population of 461,427 inhabitants in 1994 and 542,912 inhabitants in 2004 [19]. Its Municipal Human Development Index (MHDI) was 0.733 in 2000 and 0.800 in 2010, moving from the 32nd to the 40th place in the national ranking [20]. SL is the capital of the Maranhão State, with a population of 781,068 inhabitants in 1997 and 957,515 inhabitants in 2007 [21]. It is located in the Northeast, one of the poorest regions in the country, and had an MHDI of 0.658 in 2000 and 0.768 in 2010, climbing from the 516th to the 249th place in the national ranking [20]. The *per capita* income of RP is twice that of SL [22].

The RP cohort consisted of 2,846 liveborns delivered by mothers residing in the municipality at the 10 city hospitals from April to August 1994, representing 99% of all live births during the period. Losses corresponded to less than 5% of births [17]. The SL cohort consisted of 2,541 newborns selected by systematic sampling. One of each seven births in the 10 hospitals of the city was selected at random from March 1997 to February 1998. Losses occurred in 5.8% of cases. The sample was representative of childbirths in the city, as hospital deliveries represented 96.3% of all city births [18]. In both cities, losses refer to children whose mothers refused to participate in the study or who had been discharged from hospital before the data collection team arrived. Data were collected in a similar manner in both cities using a standardized questionnaire applied to the puerperae immediately after birth in order to obtain demographic and socioeconomic data, as well as information regarding pregnancy and delivery, including gestational age and newborn weight. Birth weight was measured by trained personnel using a baby scale [17,23].

Samples of the two cohorts were reevaluated at school age, with the subjects being divided into five birth weight groups: very low birth weight (VLBW, birth weight of less than 1,500 g), low birth weight (LBW, birth weight of 1,500 to 2,499 g), insufficient birth weight (IBW, birth weight of 2,500 g to 2,999 g), normal birth weight (NBW, 3,000 to 4,249 g), and high birth weight (HBW, birth weight ≥ 4,250 g, children whose birth weight was at least two standard deviations above the population mean). Children in the weight groups including a smaller number of newborns (VLBW, LBW, HBW) were oversampled in order to increase the study power. At reevaluation, RP children were 10 and 11 years old and SL children were seven to nine years old. The children in the two cities were searched and located in the schools. All parents or persons responsible for VLBW, LBW and HBW children and for one in each three children in the IBW and NBW groups were invited to participate in the study by telephone or mail. In SL, after exclusion of multiple births (n = 50), stillborns (n = 48) and infants who died in the first year of life (n = 65), 926 children were eligible for follow-up. Of these, 673 were evaluated, five of them being VLBW, 76 LBW, 19 HBW, 134 IBW, and 439 NBW, representing a follow-up rate of 72.7% [23].

In RP, after exclusion of multiple births (n = 65) and infants who died in the first year of life (n = 48), 1,150 were eligible for follow-up. Of these, 790 were evaluated, 24 of them being VLBW, 145 LBW, 174 IBW, 419 NBW, and 28 HBW [23].

Sample size calculations were performed in Epi-Info software, version 7.0 (CDC, Atlanta, USA). A sample of 676 children in each city has an 80% power to detect significant prevalence ratios (PR) equal to or higher than 1.22, considering a 50% prevalence of PNNSH and a 1:1 ratio between exposed and unexposed individuals, with a 5% probability of type I error. Furthermore, a sample of 637 children in each city has an 80% power to detect significant prevalence ratios (PR) equal to or higher than 1.38, considering a 50% prevalence of PNNSH and a 1:9 ratio between exposed and unexposed individuals, with a 5% probability of type I error.

In addition, power calculations were performed in Stata 13.0 software (Stata Corporation, College Station, USA) using data from São Luís because the sample size there was smaller than in Ribeirão Preto. Contrasting 81 low birth weight children with their 592 non-low birth weight counterparts, the power to detect significant prevalence ratios (PR) equal to or higher than 1.38, considering a 50% prevalence of PNNSH, with a 5% probability of type I error was 89.49%. Contrasting 115 children with breast feeding duration ≥12 months with 275 children breastfed for <6 months, the power to detect significant prevalence ratios (PR) equal to or higher than 1.38, considering a 50% prevalence of PNNSH, with a 5% probability of type I error was 87.69%.

The children were reevaluated at school age by means of a standardized questionnaire applied to the parents or persons responsible for them, containing questions about child’s social, demographic, general and oral health characteristics.

The dependent variable, PNNSH, was defined as pacifier and/or finger sucking maintained for 36 months of age or more [9,24]. The questionnaire asked whether the child ever had the habit to suck a pacifier and/or a finger and at what age the habit was dropped.

The independent variables collected at birth and used for analysis were: maternal age (in completed years), maternal schooling (in completed years of study), monthly family income (minimum wages), maternal marital status (married, in consensual union, without a companion), maternal smoking during pregnancy (yes if she smoked at least one cigarette a day), mother’s work outside the home (yes and no), and parity (1, 2 to 4, ≥5 children), preterm birth (yes if gestational age was less than 37 weeks estimated by the date of the last menstrual period), LBW (yes if <2500 g) and child’s sex. The following variables were obtained at school age: duration of breast-feeding (<6, 6–11 and ≥12 months), duration of bottle feeding (<12, 12–23 and ≥24 months) and current occupation of the family head (non-manual, skilled manual, unskilled manual).

Due to the oversampling of VLBW, LBW and HBW cases, the estimates were corrected by weighting using the variables "birth weight" and "preterm birth". Complex sample design was considered in all statistical analyses (sampling stratified by birth weight and application of sampling weights) [23].

The associations between the dependent variable and the remaining variables were determined by the chi-square test with α = 0.05. Crude and adjusted prevalence ratios (PR) with their respective 95% confidence intervals (95%CI) were calculated by Poisson regression with robust adjustment of variance since the prevalence of PNNSH was more than 10% in both cities [25]. We tested for the presence of collinearity using the command _rmcoll in Stata.

Two models were fitted: Model 1 was adjusted for birth variables and Model 2 included birth and school age variables. The tables show the unweighted absolute frequencies and the weighted proportions of the variables. Each variable presented percentage of missing values <5% in both cities. So, we performed analyses excluding missing values. The Stata 9.0 package was used for statistical analysis.

At birth, consent for the study was requested from the hospitals’ directors and mothers were interviewed after giving informed verbal consent. At school age, the parents or persons responsible for the children signed the informed consent form after reading the plain language statement. The study was approved by the Research Ethics Committees of the University Hospital, Ribeirão Preto Medical School, University of São Paulo (HCFMRP-USP) and of the University Hospital of the Federal University of Maranhão (HU-UFMA).

## Results

The prevalence of PNNSH differed significantly between the two cities, being higher in the more developed city – RP (47.9%; n = 372; 95%CI: 44.4%-51.5%) than in the less developed one – SL (20.2%; n = 132; 95%CI: 17.2%-23.5%). Missing data regarding PNNSH was observed in 14 subjects in RP and 19 in SL.

The percentage of teenage mothers was higher in SL (29.3%) than in RP (16.6%) whereas the percentage of maternal age ≥35 years was higher in RP (11.5%) than in SL (4.6%). Low schooling (≤4 years of study) was observed in 21.6% of RP mothers and in 15.9% of SL mothers. However, a higher percentage of high (>11 years) schooling level was observed in RP (13.1%) compared to SL (1.8%). Percentage of children from families earning ≥6 minimum wages per month was higher in RP (44.2%) than in SL (19.9%). While in RP 2/3 of the mothers were married, consensual union predominated in SL. The prevalence of smoking during pregnancy was almost five-fold higher in RP than in SL. More than 1/3 of RP mothers reported that they worked outside the home, compared to 1/5 of SL mothers. The proportion of five children or more was quite close in the two cities, and being an only child occurred in 40.3% of cases in RP and in 46.5% in SL. Preterm birth (14.9% in RP and 8.7% in SL) and LBW rates (10.6% in RP and 5.8% in SL) were higher in RP than in SL. Boys represented a little more than half the sample in both cities (Table 1).

**Table 1:** Characteristics of mothers and children at birth (frequency and weighted percentage)

**Variables**	**RP/ 1994**		**SL/ 1997/98**		P value***
	n (790)*	wtd%**	n (673)*	wtd%**	
**Maternal age (years)**					**< 0.001**
<20	131	16.6	199	29.3	
20-34	563	71.9	442	66.2	
≥35	94	11.5	32	4.6	
**Maternal schooling (years of study)**					**<0.001**
≥12	93	13.1	14	1.8	
9-11	170	22.8	255	37.8	
5 to 8	304	42.5	301	44.5	
≤ 4	158	21.6	103	15.9	
**Family income (minimum wages)**					**<0.001**
0-1	38	6.1	154	24.5	
2-3	151	26.2	243	38.8	
4-5	127	23.5	103	15.6	
≥6	240	44.2	128	19.9	
**Maternal marital status**					**<0.001**
Married	489	65.5	199	30.2	
Consensual union	158	20.8	314	46.2	
No companion	106	13.7	160	23.6	
**Maternal smoking during pregnancy**					**<0.001**
Non-smoker	595	80.3	645	96.2	
Smoker	160	19.7	28	3.8	
**Maternal work outside the home**					**<0.001**
No	469	62.0	533	78.9	
Yes	287	38.0	140	21.1	
**Parity (number of children)**					0.071
5 or more	46	5.7	36	5.2	
2 to 4	423	54.0	321	48.3	
1	313	40.3	316	46.5	
**Preterm birth**					**<0.001**
No	604	85.1	586	91.3	
Yes	186	14.9	87	8.7	
**Low birth weight**					**<0.001**
No	621	89.4	592	94.2	
Yes	169	10.6	81	5.8	
**Child’s sex**					0.724
Male	402	50.8	348	51.7	
Female	388	49.2	325	48.3	

*numbers may not add to total because missing values from same variables were excluded.

**wtd%: weighted percentage. ***Chi-square test.

Ribeirão Preto (RP), 1994, São Luís (SL), 1997/1998. Significant values are in bold.

Most children in both cities received maternal milk for a short time (<6 months) and the proportion of bottle-fed children during 24 months or more was expressive, especially in RP (59.4%). In RP, 43.4% of the family heads were unskilled manual workers, as compared to 58.0% in SL (Table 2).

**Table 2:** Characteristics of the children obtained at school age (frequency and weighted percentage)

**Variables**	**RP/ 2004/05**		**SL/ 2004/05**		P Value***
	n*(790)	wtd%**	n*(673)	wtd%**	
**Duration of breast-feeding (months)**					**0.017**
< 6	374	46.7	275	42.4	
6-11	229	32.3	260	39.8	
≥ 12	152	21.0	115	17.8	
**Duration of bottle feeding (months)**					**<0.001**
< 12	168	22.6	385	61.5	
12-23	136	18.0	128	21.0	
≥ 24	443	59.4	110	17.5	
**Current occupation of the family head**					**<0.001**
Non-manual	150	19.1	85	12.1	
Skilled manual	296	37.5	200	29.9	
Unskilled manual	340	43.4	384	58.0	
**Child’s age (years)**					**<0.001**
11	473	55.7	-	-	
10	317	44.3	-	-	
9	-	-	98	14.4	
8	-	-	572	85.2	
7	-	-	3	00.4	

*numbers may not add to total because missing values from same variables were excluded.

**wtd%: weighted percentage. ***Chi-square test.

Ribeirão Preto (RP) and São Luís (SL), 2004/05.

Table 3 shows that in RP children who were fed human milk presented lower prevalence of PNNSH in both non-adjusted and adjusted analysis. Those bottle fed for 24 months or more had a more than two-fold higher prevalence of PNNSH compared to those bottle fed for six months or less, even after adjustment for school age variables in Model 2. Girls had a 27% higher prevalence of PNNSH than boys in Model 2. The remaining birth and school age variables were not associated with PNNSH.

**Table 3:** Non-adjusted analysis and analysis adjusted by the Poisson regression model of the factors associated with prolonged non-nutritive sucking habits among Ribeirão Preto schoolchildren (2004/05)

**Variables**	**PNNSH**		**PR (95% CI)**	**PR (95% CI)**	**PR (95% CI)**
	**n**	**(wtd%)**	**(Non-adjusted)**	**(Model 1)**	**(Model 2)**
**Maternal age**					
<20	259	51.75	1.10(0.90-1.35)	1.12(0.88-1.43)	1.15(0.93-1.44)
20-34	45	46.84	1.00	1.00	1.00
≥35	68	47.49	1.01(0.79-1.30)	0.97(0.74-1.27)	1.19(0.93-1.53)
**Maternal schooling (years of study)**					
≥ 12	43	49.38	1.00	1.00	1.00
9-11	77	46.35	0.94(0.71-1.25)	0.96(0,71-1.30)	0.95(0.69-1.30)
5 to 8	147	48.90	0.99(0.77-1.28)	1.00(0.75-1.33)	0.96(0.70-1.35)
≤ 4	70	44.54	0.90(0.67-1.21)	0.91(0.66-1.27)	0.90(0.62-1.32)
**Maternal marital status**					
Married	225	46.59	1.00	1.00	1.00
Consensual union	71	45.28	0.97(0.79-1.20)	0.96(0.75-1.22)	0.99(0.78-1.26)
No companion	54	51.08	1.10(0.87-1.38)	1.07(0.83-1.38)	1.04(0.81-1.33)
**Maternal smoking during pregnancy**					
Non-smoker	267	45.38	1.00	1.00	1.00
Smoker	84	53.51	1.18(0.98-1.42)	1.20(0.98-1.46)	1.13(0.93-1.36)
**Maternal work outside the home**					
No	211	45.90	1.00	1.00	1.00
Yes	141	48.89	1.06(0.90-1.26)	1.10(0.91-1.33)	1.05(0.87-1.27)
**Parity (number of children)**					
5 or more	24	51.57	1.12(0.81-1.55)	1.04(0.70-1.55)	1.12(0.79-1.60)
2 to 4	189	46.16	1.00	1.00	1.00
1	154	48.16	1.04(0.88-1.23)	0.93(0.76-1.13)	0.89(0.74-1.08)
**Preterm birth**					
No	276	46.94	1.00	1.00	1.00
Yes	96	52.16	1.11(0.94-1.32)	1.06(0.86-1.29)	0.90(0.74-1.10)
**Low birth weight**					
No	282	46.81	1.00	1.00	1.00
Yes	90	54.52	1.16(0.99-1.38)	1.03(0.84-1.27)	1.05(0.86-1.30)
**Child’ sex**					
Male	176	44.33	1.00	1.00	1.00
Female	196	50.91	1.15(0.98-1.35)	**1.24(1.04-1.47)**	**1.27(1.08-1.51)**
**Current occupation of the family head**					
Non-manual	67	46.15	1.00		1.00
Skilled manual	142	48.17	1.04(0.83-1.31)		1.13(0.87-1.47)
Unskilled manual	162	48.17	1.04(0.84-1.30)		1.13(0.85-1.50)
**Duration of breast-feeding (months)**					
< 6	205	56.33	1.00		1.00
6-11	112	49.79	0.88(0.75-1.05)		0.91(0.76-1.09)
≥ 12	41	26.04	**0.46(0.34-0.62)**		**0.53(0.37-0.74)**
**Duration of bottle feeding (months)**					
< 12	44	23.63	1.00		1.00
12-23	44	34.42	1.46(0.99-2.14)		1.30(0.86-1.97)
≥ 24	262	60.53	**2.56(1.90-3.45)**		**2.24(1.60-3.14)**

n = Number of participants. Significant values are in bold.

PNNSH (wtd%) = Prolonged non-nutritive sucking habits (weighted prevalence).

PR (95% CI) = Weighted estimate of the prevalence ratio (95% confidence interval) – non-adjusted model.

PR (95% CI) (Model 1) = Weighted Poisson regression model adjusted for birth variables.

PR (95% CI) (Model 2) = Weighted Poisson regression model adjusted for birth and school age variables.

In SL (Table 4), non-adjusted analysis showed that bottle and breast feeding were associated with PNNSH. Children bottle fed for ≥24 months had a nearly three-fold higher prevalence of PNNSH compared to those bottle fed for <12 months. Children breast fed for ≥ 12 months had a much lower prevalence of PNNSH (PR = 0.36, 95% CI 0.20-0.66) than those who received breast milk for <6 months. These associations attenuated a little but persisted after adjustment for birth and school age variables (Model 2). Both adjusted models showed that PNNSH were more prevalent among girls than boys, as well as among children whose mothers worked outside the home. Children whose mothers lived in consensual union had a higher prevalence of PNNSH than children whose mothers were married, but this association occurred only in Model 2. Collinearity was not detected in any of the multivariate analyses.

**Table 4:** Non-adjusted analysis and analysis adjusted by the Poisson regression model of the factors associated with prolonged non-nutritive sucking habits among São Luís schoolchildren (2004/05)

**Variables**	**PNNSH**		**PR (95% CI)**	**PR (95% CI)**	**PR (95% CI)**
	**n**	**(wtd%)**	**(Non-adjusted)**	**(Model 1)**	**(Model 2)**
**Maternal age**					
<20	41	20.34	0.98(0.70-1.37)	1.04(0.69-1.55)	1.17(0.79-1.73)
20-34	88	20.79	1.00	1.00	1.00
≥35	3	11.09	0.53(0.18-1.58)	0.43(0.17-1.12)	0.35(0.10-1.23)
**Maternal schooling (years of study)**					
≥ 12	2	16.98	1.00	1.00	1.00
9-11	57	22.02	1.30(0.36-4.64)	1.39(0.42-4.58)	1.79(0.55-5.87)
5 to 8	55	19.02	1.12(0.31-4.01)	1.23(0.37-4.08)	1.60(0.48-5.33)
≤ 4	18	19.57	1.15(0.31-4.31)	1.25(0.36-4.31)	2.15(0.60-7.69)
**Maternal marital status**					
Married	33	15.78	1.00	1.00	1.00
Consensual union	64	21.62	1.37(0.93-2.02)	1.45(0.98-2.16)	**1.62(1.07-2.44)**
No companion	35	23.21	1.47(0.95-2.28)	1.36(0.87-2.13)	1.61(0.93-2.43)
**Maternal smoking during pregnancy**					
Non-smoker	128	20.32	1.00	1.00	1.00
Smoker	4	17.71	0.87(0.36-2.13)	0.98(0.40-2.41)	1.32(0.51-3.41)
**Maternal work outside the home**					
No	95	18.18	1.00	1.00	1.00
Yes	37	27.93	**1.54(1.10-2.14)**	**1.63(1.14-2.33)**	**1.51(1.03-2.22)**
**Parity (number of children)**					
5 or more	6	20.10	1.08(0.51-2.28)	1.42(0.70-2.86)	1.03(0.39-2.70)
2 to 4	58	18.58	1.00	1.00	1.00
1	68	21.90	1.18(0.86-1.62)	1.06(0.73-1.53)	0.99(0.68-1.43)
**Preterm birth**					
No	111	19.59	1.00	1.00	1.00
Yes	21	25.05	1.28(0.84-1.95)	1.23(0.78-1.93)	1.47(0.98-2.21)
**Low birth weight**					
No	115	20.07	1.00	1.00	1.00
Yes	17	22.20	1.10(0.70-1.75)	0.99(0.60-1.63)	0.92(0.57-1.48)
**Child’ sex**					
Male	57	17.21	1.00	1.00	1.00
Female	75	23.36	1.36(0.99-1.86)	**1.40(1.02-1.91)**	**1.47(1.06-2.02)**
**Current occupation of the family head**					
Non-manual	19	23.47	1.00		1.00
Skilled Manual	43	20.79	0.89(0.54-1.44)		0.78(0.48-1.27)
Unskilled manual	70	19.47	0.83(0.53-1.30)		0.67(0.42-1.09)
**Duration of breast feeding (months)**					
< 6	74	27.55	1.00		1.00
6-11	44	17.41	**0.63(0.45-0.89)**		**0.66(0.47-0.95)**
≥ 12	11	9.90	**0.36(0.20-0.66)**		**0.31(0.17-0.59)**
**Duration of bottle feeding (months)**					
< 12	48	13.09	1.00		1.00
12-23	34	25.96	**1.98(1.33-2.96)**		**1.96(1.28-2.98)**
≥ 24	40	36.56	**2.79(1.93-4.03)**		**2.49(1.70-3.63)**

n = Number of participants. Significant values are in bold.

PNNSH (wtd%) = Prolonged non-nutritive sucking habits (weighted prevalence).

PR (95% CI) = Weighted estimate of the prevalence ratio (95% confidence interval) – non-adjusted model.

PR (95% CI) (Model 1) = Weighted Poisson regression model adjusted for birth variables.

PR (95% CI) (Model 2) = Weighted Poisson regression model adjusted for birth and school age variables.

## Discussion

The prevalence of PNNSH was high in both cities, being 2.4 times higher in RP than in SL. The factors associated with prevalence of PNNSH differed between the two cohorts. In RP, female sex and bottle feeding for ≥24 months, and in SL female sex, bottle feeding for ≥12 months, maternal work outside the home and having parents living in consensual union were factors associated with a higher prevalence of PNNSH. Breast feeding for ≥12 months in RP and ≥6 months in SL was associated with lower prevalence of PNNSH. None of the perinatal factors investigated – preterm birth, birth weight and maternal age at childbirth – were associated with PNNSH.

The prevalence of PNNSH in RP was similar to that observed by Heimer, Katz and Rosenblatt [8] and higher than that observed in SL. The prevalence of NNSH varies widely in the international literature from 3% to 63.2% [5,11,14,15,26,27] as well as in Brazil, from 13.8% to 67% [15,28-30]. This variability may be due to cultural differences, different age ranges [31] and to the different methods used in the estimation of PNNSH. Some studies have evaluated the use of a pacifier only [11,12] or finger sucking only [26,32] and most of them used a cross-sectional design without taking into account lifelong prevalence or duration of the habit, as done in the present study. In addition, because of the availability of better technology, more preterm and LBW babies were born in RP than in SL [33] and received earlier artificial feeding [34], a fact that may have contributed to the development of PNNSH. Furthermore, a recent study on the frequency of breast-feeding in Brazil [35] reported that SL children had the highest probability to be receiving exclusive breast-feeding at six months of age (12.5%) among capital cities of the Brazilian Northeast, and the third highest probability for the country, only behind Belém (13.3%) and Florianópolis (13.1%). This higher frequency of breast-feeding may help explain the lower prevalence of PNNSH in this city compared to RP.

In RP, the more developed city, the families have a higher income and educational level and there is a greater proportion of married mothers, although there is also a higher proportion of mothers who are smokers and who work outside the home compared to SL mothers. Thus, considering the evidence that socioeconomic factors such as maternal employment may affect the psychological status of the child [12], and that this may manifest as oral non-nutritive habits like pacifier and finger sucking, the differences observed are plausible, although in the present study an association between maternal employment and PNNSH was only observed in the less developed city, SL. Some studies have demonstrated that a mother’s job causes a distance between mother and children, who look for emotional compensation by keeping oral habits after three years of age. Also, lack of information and low access to health care predispose to the maintenance of these habits. The response of a child to separation from his/her mother may manifest in the form of regressive habits, mechanisms of compensation for the sensations of insecurity [12,16,29].

There is no consensus about the influence of maternal schooling and family income on the occurrence of PNNSH. Santos et al. [15] observed a tendency to the persistence of pacifier sucking among children of mothers with a high educational level. In contrast, Tomita et al. [16], Stone et al. [5] and Heimer et al. [8] observed a lower frequency of pacifier sucking among children of mothers with higher educational level. In a study conducted in the Brazilian Southeast [16], family income was not significantly associated with NNSH, whereas Santos et al. [15] recorded a higher frequency of pacifier sucking and a lower frequency of finger sucking among children belonging to higher income families. In the present study, among the socioeconomic factors evaluated, only marital status, and only in SL, was found to be associated with a higher prevalence of these habits, being higher among children whose parents live in consensual union. Although the proportion of PNNSH was about 30% higher among children of mothers without a companion than among children of married mothers, this difference was not statistically significant. Santos et al. [15], in a study on another city in the Brazilian Northeast, did not observe an association between these habits and mother’s marital status. In a study on this same cohort in SL, a higher prevalence of emotional symptoms was observed among children of mothers without a companion or living in consensual union [36], a fact that may explain in part the higher prevalence of these deleterious habits in these children.

The prevalence of PNNSH was higher among girls in both cities, in agreement with another study [16] that observed a higher prevalence of NNSH in the upper class and among girls. There is no consensus on these findings in the literature. Some studies reported a higher frequency of these habits among girls [29], others reported a higher frequency among boys [30], and some find no difference between genders [14]. Some authors have suggested that girls have a greater tendency to the development of these habits due to the fact that they have more emotional problems than boys [15,29], but this fact was not observed in the analysis of factors associated with emotional symptoms in the children of this same cohort [36].

Several studies have associated the discontinuation of breast-feeding or a lower exposure to it and the use of a bottle with the development of finger or pacifier sucking [10,11,13,29,37-39]. In both cities there was an association between shorter breast-feeding duration, longer bottle feeding duration and higher prevalence of PNNSH. The relations between these variables have not been fully clarified. The results of a systematic review of the literature indicates that the association between breast-feeding and NNSH is detected only in observational studies, but not in studies with a higher level of evidence, such as randomized clinical trials [40]. A meta-analysis of observational studies has suggested that the use of a pacifier is associated with reduction or cessation of breast-feeding [39].

There are some proposed mechanisms for the association between pacifier use and reduced breast-feeding. Firstly, "*nipple confusion*" that is the term commonly applied to mechanical differences between suckling at the breast and sucking on a pacifier or bottle nipple, may impair breast-feeding [41]. Infants that are using pacifiers tend to suck less on the breast, and as a result this reduces the milk supply, subsequently ending breast-feeding [39]. On the other hand, sucking on the mother’s breast supplies nutritional and emotional needs [42]. So, children who are not breast fed, but fed on a bottle, may have unmet suction and psycho-affective needs and use a pacifier to satisfy them. Although this association may not be causal, there is some evidence that this form of feeding at the beginning of life may affect the persistence of deleterious oral habits, such as PNNSH.

In this study, no association was observed between maternal smoking during pregnancy and PNNSH. Few studies have investigated this association. Only Stone et al. [5], in a cohort study, observed that mothers who had been smokers offered more pacifiers to their children. However, in contrast to the present study, the authors evaluated NNSH only at 15 months of age without referring to prolonged sucking and information about maternal smoking was obtained at the same time at which NNSH data were abstracted.

A study conducted on 52 Brazilian children aged 2–5 years detected a higher prevalence of pacifier sucking among preterm children and children of extremely low birth weight [43]. Early weaning appears to be more common among VLBW children [44], a fact that may increase the risk of these children to develop NNSH. However, in the present study, neither birth weight nor preterm birth were associated with PNNSH.

One of the limits of the present study was the impossibility to determine whether infants fed human milk received it by breast-feeding or in a bottle, cup or spoon. However, receiving human milk by cup or spoon is not a widespread practice worldwide, as well as in Brazil. Only a very small percentage of children receive human milk by spoon or cup, especially children of HIV positive women and extremely underweight children who are fed human milk from human milk bank, but only for a short period of time in their lives [45]. Furthermore, a randomized clinical trial analyzing associations between cup feeding, pacifier or bottle-feeding use and breastfeeding showed that supplementary feedings, regardless of the method (cup or bottle) used, have a detrimental effect on breast-feeding duration. There were no differences in cup versus bottle use on breast-feeding duration [41]. Among babies under neonatal intensive care, a study in Italy showed that 30.5% of the sample was exclusively breastfed and only 10% sucked directly at the breast. Human milk feeding using bottle, cup, spoon or other strategy had a low prevalence among babies under neonatal intensive care [46]. Thus, not having this information is not a crucial limitation, given that the prevalence of this habit is very low in developing countries [47]. However, even with this limitation in mind, it was possible to observe that human milk feeding was associated with lower prevalence of PNNSH in both cities.

In RP, a lower percentage of children who participated in the follow-up study were children of mothers who lived in consensual union, who were younger than 20 years and who had less than four years of schooling, compared to children who did not participate. There were no differences in the follow-up rate regarding parity or child’s sex. In SL, follow-up rate was lower among mothers of high educational level (≥12 years of study), who were primiparae or who gave birth to boys compared to the eligible group that did not participate. There was no difference between participants and non-participants regarding maternal age or marital status. Differences in birth weight and preterm birth were observed due to the study’s complex sampling design and were corrected by weighting [23].

Recall errors or telescoping effect may have been present because feeding habits and PNNSH were recorded retrospectively. Interactions terms were not added to the models. Sample size calculation and power estimation did not take into consideration the weighted estimates derived according to "birth weight" and "preterm birth" and were based on the chi-square test and not on Poisson regression. Thus the real study power might have been somewhat lower than estimated.

The present study is highly relevant by investigating a topic that has not been fully elucidated yet, by being a population-based cohort study involving two socioeconomically contrasting regions and due to the fact that VLBW, LBW and HBW were oversampled, a fact that increased the study power. In addition, it revealed that some factors commonly reported to be associated with finger and pacifier sucking at some time in life, such as maternal schooling [15,16], maternal smoking [5] and occupation of the family head [16] were not associated with PNNSH at more advanced ages, suggesting that the factors that induce the establishment of these habits may not be maintained or may differ from those that favor maintenance of these habits for a longer period of time.

## Conclusions

The prevalence of PNNSH was high, especially in the more developed location, and perinatal variables (birth weight, preterm birth and maternal age at infant’s birth) were not associated with it. Shorter breast-feeding duration and longer bottle feeding duration were consistently associated with a higher prevalence of PNNSH. Nutritional suckling feeding habits at the beginning of life seem to be predictors of PNNSH at school age.

## Competing interests

The authors certify that they have no commercial or associative interest that represents a conflict of interest in connection with the manuscript.

## Authors’ contribution

MMN, CSAF and FFPF analyzed the data and wrote the manuscript. AAMS, MCPS, MAB and HB conceived the research questions of the study, supervised the data collection of the study and were involved in critical revisions. Besides, they had full access to all of the data in the study and take responsibility for the integrity of the data and the accuracy of the data analysis. EBAFT was involved in critical revisions and data analysis. All authors have read and approved the final version of the manuscript.

## Pre-publication history

The pre-publication history for this paper can be accessed here:

http://www.biomedcentral.com/1471-2458/14/743/prepub
